# Editorial: Imaging Technology in Oncology Pharmacological Research

**DOI:** 10.3389/fphar.2021.711387

**Published:** 2021-07-23

**Authors:** Qi Zeng, Xu Cao, Jinchao Feng, Hong Shan, Xueli Chen

**Affiliations:** ^1^ Engineering Research Center of Molecular and Neuro Imaging of the Ministry of Education, School of Life Science and Technology, Xidian University, Xi’an, China; ^2^ Thayer School of Engineering, Dartmouth College, Hanover, NH, United States; ^3^ Faculty of Information Technology, Beijing University of Technology, Beijing, China; ^4^ Department of Interventional Medicine, Guangdong Provincial Engineering Research Center of Molecular Imaging, The Fifth Affiliated Hospital of Sun Yat-Sen University, Zhuhai, China

**Keywords:** imaging technology, oncology, pharmacological research, cancer therapy, small molecule drugs

Imaging technology is being recognized as an important tool for breaking through the bottleneck of drug development as it is able to provide great insights into the morphology or functionality of pharmacological models, including cell, tissue, and animal. Imaging has several advantages compared to traditional evaluation methods, which include high spatiotemporal resolution, imaging sensitivity, and tissue specificity. In addition, imaging can be utilized to determine the gene expression, metabolism of various substances, cancer detection, drug development, as well as other fields.

Imaging technology includes both microscopic and macroscopic imaging scales, and encompasses fluorescence-based microscopy (Weissleder and Pittet, 2008), Raman-based microscopy (Chen et al., 2017), targeted ultrasound imaging (Frangioni, 2008), photoacoustic imaging (Wang and Hu, 2012), SPECT, PET (Rahmim and Zaidi, 2008), and molecular MRI (Pichler et al., 2008), among others. These technologies can be utilized for imaging or analysis of living systems at various levels. Hence, imaging strategies are commonly applied across several research projects to evaluate the pharmacokinetics, activity, and mechanisms of cancer therapy through the use of small molecule drugs or prodrugs (Gillies, 2002; Wang et al., 2008; Gurny and Mader, 2010; Janib et al., 2010). In parallel, more specific imaging-based evaluation methods have been developed that can help improve the pharmacological studies of chemotherapy. Thus, in this special issue, we further emphasize and describe the research that has provided creative research ideas in this domain.

Two review articles demonstrated the role of one imaging technology in the treatment of cancer. Fang et al. systemically summarized the recent advances that have been made in the development of noninvasive imaging and radiotherapy agents for the different molecular subtypes of breast cancer in preclinical studies in their study, “Theranostics for the different molecular subtypes of breast cancer.” The researchers provided a conceptual examination of recent or current original articles that were published within the last decade in the field of preclinical breast cancer nuclear imaging. Data were extracted from the PubMed database and filtered according to the key words “breast cancer,” “preclinical,” “PET/SPECT,” and “targeted imaging.” In order to help guide future investigations of novel theranostic agents, they listed different imaging agents and cell lines that were tested in preclinical studies. They think molecular imaging can help with diagnosis, staging, guiding treatment, and predicting response to corresponding targeted therapy. The review of Zeng et al. which was titled “Treatment coherent Raman scattering microscopy in oncology pharmacokinetic research” highlighted coherent Raman scattering (CRS) microscopy as a novel emerging platform to facilitate oncology pharmacokinetic research. It would be of great importance to develop label-free optical microscopy that is able to assess stability and dissolution of drugs in the solid state, and uptake, distribution, interaction, and excretion of anticancer drug nanocarriers in a biological environment. Therefore, they summarized the recent technical advances and applications of CRS microscopy in the field of anticancer drug pharmacokinetics at the single cell level, drug stability and dissolution in the solid state, as well as the activities of anticancer drug nanocarriers in single cells. According to their review, there are several reasons to believe that CRS microscopy with label-free, chemically selective, high temporospatial resolution, and highly sensitive imaging can offer novel opportunities for investigation of anticancer drugs.

In order to explore a novel strategy of cancer treatment, imaging technology can be applied across several *in vitro* studies. Zhang et al. in the article “Metabolic reprogramming of sulfur in hepatocellular carcinoma and sulfane sulfur-triggered anti-cancer strategy” uncovered reprogramming of sulfur metabolism in hepatocellular carcinoma (HCC) and were able to provide a potential therapeutic strategy for HCC by donating sulfane sulfurs. Herein, the cell imaging assay was carried out to support their hypothesis. Their findings suggest that application of sulfane sulfurs may be an effective therapeutic strategy, particularly for HCC tumor cells that have reprogrammed the sulfur metabolism. Jin et al. in their article “Hirsutella sinensis fungus regulates CD8 + T cell exhaustion through involvement of T-Bet/Eomes in the tumor microenvironment” have provided insights into the application of Hirsutella sinensis fungus (HSF) in a tumor immune treatment. Their study demonstrated that HSF exerts antitumor effects mainly by reducing the expression of immune checkpoints by inhibiting T-bet in T cells, which lowers Tex cell production in the tumor microenvironment. Additionally, HSF could promote the Eomes expression in order to enhance T cell function. *In vivo* imaging technology was utilized to evaluate the effects of HSF on various tumor mouse models. Their findings were able to provide novel insights into the effect of HSF on tumor immune responses.

Imaging technology was a powerful tool for the *in vivo* evaluation of drugs. Xu et al. in their article “Bioluminescence imaging-based assessment of the anti-triple-negative breast cancer and NF-Kappa B pathway inhibition activity of britanin” were able to evaluate the anti–breast cancer activity of britanin. Their results demonstrated that britanin induces apoptosis *via* inhibition of the NF-κB pathways. The bioluminescence imaging screening system is useful for accelerating application of britanin in the antitumor field, which provides a useful tool for evaluating the efficacy of phytochemicals in inhibiting cancer cell proliferation in animal models. Zhan et al. in their article “Construction of biocompatible dual-drug loaded complicated nanoparticles for *in vivo* improvement of synergistic chemotherapy in esophageal cancer” developed a doxorubicin and *β*-elemene–loaded mesoporous silica nanoparticle system to exert inhibitory effects in esophageal cancer treatment. Fluorescence images were applied in order to validate efficacy of the combination therapy *in vivo*. Zhang et al. in their article “Terphenyllin suppresses orthotopic pancreatic tumor growth and prevents metastasis in mice” reported a novel marine-derived natural product terphenyllin with potent anti-pancreatic cancer (PC) activity. Herein, terphenyllin was found to significantly suppress PC cell growth and metastasis *in vitro* and *in vivo*. Terphenyllin induced PC cell apoptosis by increasing the expression of proapoptotic proteins and decreasing the expression of antiapoptotic proteins. The Panc1-Luc cell lines were utilized to develop an orthotopic mouse model, which may be able to closely mimic the original situation in human PC patients and may be better able to predict the therapeutic efficacy of the test compound. The *in vivo* imaging technique demonstrated significant inhibitory effects of terphenyllin on tumor growth. Their results reveal the therapeutic potential of terphenyllin in PC, which can help provide a basis for further developing this natural compound as an anticancer therapeutic agent.

The integrated diagnosis and treatment of nanoparticles will provide precise information for a cancer treatment strategy. In Li et al.’s “Manganese-based targeted nanoparticles for postoperative gastric cancer monitoring via magnetic resonance imaging,” an Mn-based contrast agent for MRI was synthesized to provide accurate evaluation of therapeutic effects and guide treatment strategy adjustment over time. A series of *in vitro* and *in vivo* imaging experiments were employed to assess the characters of Mn_3_O_4_@PEG-RGD NPs. Their results indicated that Mn_3_O_4_@PEG-RGD NPs likely have a great potential for the MRI postoperative monitoring of gastric cancer and give an appropriate strategy for following chemotherapy. Xu et al. in their research “Synthesis, characterization, cellular uptake, and *in vitro* anticancer activity of fullerenol-foxorubicin conjugates alpha 3 function by colchicines” designed and synthesized the fuller enol (FU)-DOX conjugates and folic acid (FA)-grafted FU-DOX conjugates in order to improve the selectivity and activity of DOX in cancer cells. They synthesized DOX and FU conjugates (FU-DOX) through the use of the acid-sensitive hydrazone bond and were further modified by FA in order to obtain FA-FU-DOX conjugates for improving tumor-targeting effects. In their study, fluorescent microscopy was utilized to monitor cellular uptake. Indeed, FA-FU-DOX conjugates may optimize the safety and efficacy profile of DOX. Zhou et al. also wrote another review article “pH-Sensitive and long-circulation nanoparticles for near-infrared fluorescence imaging-monitored and chemo-photothermal synergistic treatment against gastric cancer” which reported photothermal–chemotherapy combined nanoparticles (PCC NPs) that have functions of chemo-photothermal synergistic therapy and continuous imaging for gastric cancer. The PCC NPs consisted of dopamine, poloxamer, DOX, and IR-820 *via* π–π stacking and demonstrated good biocompatibility both *in vitro* and *in vivo*. Their study can offer a novel postoperative treatment for gastric cancer.

Moreover, imaging technology also plays a significant role in a clinical anticancer medication strategy. Zhao et al. in their article “Accuracy of endoscopic diagnosis of helicobacter pylori based on the Kyoto classification of gastritis: a multicenter study” provided evidence of clinical accuracy and robustness of the Kyoto classification of gastritis in the Chinese population. Furthermore, they discovered that the reappearance of two indicators (unclear atrophy boundary and unclear atrophy boundary) in atrophic mucosa could help sufficiently determine the presence of *Helicobacter pylori* (*H. pylori*) infection on an endoscopic basis. Their prospective and multicenter study was based on 650 Chinese patients. In order to prevent the occurrence and development of gastric cancer (GC) early on, their study offered an important novel finding for screening of early GC based on the close relationship between *H. pylori* and GC.

In conclusion, a collection of 11 articles contributed to this research topic, which covers two reviews and nine research articles. It is important to note that these published articles cover a wide spectrum of applications of imaging technology in oncology pharmacological research, which includes exploring a novel anticancer chemotherapy strategy *in vitro*, evaluating *in vivo* anticancer effects, and benefiting the clinical diagnosis. These articles provide deep insights into methodology and applications of imaging technology. We believe that imaging technology would be increasingly welcome in oncology pharmacological research.
